# Framingham Risk Score and Alternatives for Prediction of Coronary Heart Disease in Older Adults

**DOI:** 10.1371/journal.pone.0034287

**Published:** 2012-03-28

**Authors:** Nicolas Rodondi, Isabella Locatelli, Drahomir Aujesky, Javed Butler, Eric Vittinghoff, Eleanor Simonsick, Suzanne Satterfield, Anne B. Newman, Peter W. F. Wilson, Mark J. Pletcher, Douglas C. Bauer, for the Health ABC Study

**Affiliations:** 1 Department of General Internal Medicine, Inselspital, University of Bern, Bern, Switzerland; 2 Department of Ambulatory Care and Community Medicine, University of Lausanne, Lausanne, Switzerland; 3 University Institute of Social and Preventive Medicine, University of Lausanne, Lausanne, Switzerland; 4 Cardiology Division, Emory University, Atlanta, Georgia, United States of America; 5 Department of Epidemiology and Biostatistics, University of California San Francisco, San Francisco, California, United States of America; 6 Intramural Research Program, National Institute on Aging, Baltimore, Maryland, United States of America; 7 Department of Preventive Medicine, University of Tennessee College of Medicine, Memphis, Tennessee, United States of America; 8 Department of Epidemiology, University of Pittsburgh, Pittsburgh, Pennsylvania, United States of America; 9 Emory Clinical Cardiovascular Research Institute, Atlanta, Georgia, United States of America; 10 Division of General Internal Medicine, Department of Medicine, University of California San Francisco, San Francisco, California, United States of America; FuWai hospital, Chinese Academy of Medical Sciences, China

## Abstract

**Background:**

Guidelines for the prevention of coronary heart disease (CHD) recommend use of Framingham-based risk scores that were developed in white middle-aged populations. It remains unclear whether and how CHD risk prediction might be improved among older adults. We aimed to compare the prognostic performance of the Framingham risk score (FRS), directly and after recalibration, with refit functions derived from the present cohort, as well as to assess the utility of adding other routinely available risk parameters to FRS.

**Methods:**

Among 2193 black and white older adults (mean age, 73.5 years) without pre-existing cardiovascular disease from the Health ABC cohort, we examined adjudicated CHD events, defined as incident myocardial infarction, CHD death, and hospitalization for angina or coronary revascularization.

**Results:**

During 8-year follow-up, 351 participants experienced CHD events. The FRS poorly discriminated between persons who experienced CHD events vs. not (C-index: 0.577 in women; 0.583 in men) and underestimated absolute risk prediction by 51% in women and 8% in men. Recalibration of the FRS improved absolute risk prediction, particulary for women. For both genders, refitting these functions substantially improved absolute risk prediction, with similar discrimination to the FRS. Results did not differ between whites and blacks. The addition of lifestyle variables, waist circumference and creatinine did not improve risk prediction beyond risk factors of the FRS.

**Conclusions:**

The FRS underestimates CHD risk in older adults, particularly in women, although traditional risk factors remain the best predictors of CHD. Re-estimated risk functions using these factors improve accurate estimation of absolute risk.

## Introduction

Guidelines for the prevention of coronary heart disease (CHD) recommend the use of risk scores to identify adults at higher risk of CHD for whom preventive therapy–e.g., by lipid lowering drugs–has higher absolute benefits [1]. Several scoring systems exist to help clinicians assess the 10-year CHD risk [2], [3], [4], with the Framingham risk score (FRS) [2] the most widely used. US Guidelines for the prescription of lipid-lowering drug therapy [5] and aspirin in primary prevention [6] are based on the risk estimations provided by the FRS.

Most risk scores were developed in white middle-aged populations [2], [3], [4]. Thus, it is uncertain whether risk estimates based on these scores can be generalized to the elderly. The FRS, for example, was developed in a white middle-aged population with a mean age of 49 years and included persons as young as 30 and none older than 74 [2]. Actual risk prediction with FRS might perform less well in older adults compared to middle-aged adults, and some traditional risk factors have weaker associations with CHD risk in the elderly; for example, total and LDL-cholesterol are strong cardiovascular risk factors in middle-aged but not in older adults [7].

As it remains unclear whether and how CHD risk prediction might be improved in the growing population of elderly [8] to facilitate primary prevention strategies, we aimed to compare the prognostic performance of 1) the FRS, directly and 2) after recalibration [9], and 3) with functions derived from the Health ABC Study, a cohort of elderly white and black men and women [10]. We also aimed to assess 4) the utility of adding routinely available lifestyle and simple laboratory variables not part of the FRS but which have been shown to predict CHD in older adults, such as creatinine [11], glucose [12] and lifestyle factors (alcohol consumption [13], physical activity [14]).

## Methods

### Study population

Participants were part of the Health, Aging, and Body Composition Study (Health ABC Study), a population-based cohort of 3075 community-dwelling men and women, aged 70–79 during the study enrollment period in 1997–1998. Participants were identified from a random sample of white and all black Medicare-eligible adults living in designated zip codes areas surrounding Pittsburgh, PA, and Memphis, TN. Eligibility criteria at baseline included the ability to walk ¼ mile, up 10 stairs without rest and perform basic activities of daily living independently [10]. All participants gave written informed consent and the Pittsburgh and Memphis Institutional Review Boards approved the protocol.

Among the 3075 participants, we excluded 841 who had overt cardiovascular disease (CVD) at baseline, defined as diagnosis of CHD (angina, prior myocardial infarction, angioplasty of coronary arteries or coronary artery surgery), stroke or transient ischemic attack, peripheral arterial revascularization, carotid artery disease, heart failure or having a pacemaker. We also excluded 41 participants with missing data for any of the traditional cardiovascular risk factors. The final sample for our analyses was 2193 participants.

### Measurements

#### Cardiovascular risk factors

Participants reported smoking history and were classified as never, current, or former smoker. Fasting total cholesterol, HDL-cholesterol, and blood pressure were measured as previously described [15]. Hypertension was defined as self-report and use of anti-hypertensive medications, or measured blood pressure ≥140 and/or ≥90 mm Hg. Diabetes was defined as self-reported medical diagnosis and/or using any hypoglycemic medication [16]. Physical activity was assessed by questionnaire about all types of walking and exercise performed in the prior week [14].

#### Cardiovascular events

During 8-year follow-up, we assessed incident CHD events and mortality among participants without overt CVD at baseline [16]. Using algorithms mirroring those of the Cardiovascular Health Study [16], diagnoses and cause of death were adjudicated until 2006–2007 based on interview, review of all hospital records, death certificates, and other documents by a panel of clinicians. CHD events included nonfatal myocardial infarction or coronary death (corresponding to “hard” events, as defined in the current FRS [5]), and hospitalization for angina or revascularization (coronary angioplasty or surgery) [17].

### Statistical Analyses

The FRS predicts 10-year CHD risk based on a Cox model estimated using data from the Framingham Heart Study [2]. The Framingham cohort included 5345 subjects aged 30–74 years at the time of their examination in 1971–1974. For this analysis, we used the sex-specific Framingham equations of Wilson [2], because they include diabetes, a strong independent CHD risk factor [18], [19]. This FRS Cox model includes age, total and HDL cholesterol, blood pressure, diabetes, and smoking status.

In this study, we compared the prognostic performance of the FRS, directly and after recalibration (taking into account different prevalence of risk factors and underlying rates of developing CHD), with functions entirely derived from the Health ABC cohort, similar to previous studies [9]. Analyses were stratified by gender. We first estimated the FRS using regression coefficient estimates and values of the risk factor means reported by Wilson [2]. To account for the shorter follow-up in the Health ABC study and to avoid extrapolation beyond the range of the data [17], we examined 7.5-year risk and adapted accordingly the estimated baseline survival function used in computing the FRS. Participants who died from non-CHD death were censored at the time of death.

We then examined whether the predictive performance of the FRS could be improved with recalibration or with refitting model coefficients. For the *recalibrated* version of the FRS [9], we re-estimated predicted risks for Health ABC by retaining the original coefficient estimates reported by Wilson [2] but adapted the risk factor means to the present cohort and the Kaplan Meier estimate of the baseline survival function of Health ABC data. For the *refit* version of the FRS (the “Health ABC function”), we estimated the regression coefficients with a Cox model fitted to the Health ABC data, obtaining an estimated predicted risk entirely based on Health ABC data. In this model, some adjacent risk factor categories were combined to avoid cells with limited numbers of events and/or unpredictive trends.

To compare prediction of these three risk models, we examined different statistical measures. To assess discrimination, we used Harrell's C-index [20], an adaptation of the C-statistic an adaptation of the C-statistic or area under the ROC curve for use with survival data. As the model validation for Health ABC functions was performed on the same dataset used for estimating the Cox model and the sample included too few events for split-sample validation, we calculated an optimism-corrected C-index using bootstrap resampling [21] with 1000 replications [20]. To assess model calibration, we used Parzen's adaptation [22] of the Hosmer-Lemeshow test to the Cox model.

In exploratory analysis, we sought to determine whether alternative sets of predictors would improve risk prediction. To evaluate the utility of adding to the FRS different lifestyle and simple laboratory variables, we initially considered predictor variables with p<0.20 in unadjusted Cox models for CHD events in Health ABC data. We then used three model selection procedures: a backward selection with a retention criterion of p<0.10, and two forward stepwise selection procedures minimizing the Akaike Information Criterion (AIC) and the Bayesian Information Criterion (BIC), respectively [23]. In these models, total cholesterol, HDL-cholesterol, and blood pressure were modeled as continuous predictors. Statistical analyses were performed using the software R, version 2.9.1 (R Foundation for Statistical Computing, Vienna, Austria).

## Results

At baseline, the mean age of the study participants was 73.5 years; 55% were women, and 41% were black (Table 1). The mean 10-year risk based on the FRS was 14.9%. Most participants had a 10-year CHD risk ranging from 5 to 19.9%.

**Table 1:** Baseline characteristics and unadjusted associations with incident CHD events (n = 2193; number of CHD events = 351).

Variable	Mean ± SD / n (%)	HR (95% CI)	p
Age	73.50±2.85	1.03 (1.00, 1.07)	0.09
Age (categories)			0.03*
70–71	672 (30.6)		
72–75	934 (42.6)	0.99 (0.77, 1.27)	
76–78	464 (21.2)	1.05 (0.78, 1.41)	
79	123 (5.6)	1.59 (1.04, 2.43)	
Gender			
Men	981 (44.7)		
Women	1212 (55.3)	0.52 (0.42, 0.65)	<0.001
Race			
White	1293 (59.0)		
Black	900 (41.0)	0.96 (0.78, 1.19)	0.73
Site			
Memphis	1125 (51.3)		
Pittsburgh	1068 (48.7)	0.99 (0.79, 1.22)	0.89
Education			0.29*
<high school	532 (24.3)		
High school graduate	734 (33.6)	0.81 (0.61, 1.07)	
Postsecondary	922 (42.1)	0.87 (0.67, 1.13)	
Smoking status			0.03*
Never	1016 (46.3)		
Former	956 (43.6)	1.41 (1.13, 1.77)	
Current	221 (10.1)	1.49 (1.04, 2.12)	
Alcohol, drinks/wk			0.41*
<1	1535 (70.3)		
1–7	482 (22.1)	0.88 (0.68, 1.14)	
>7	166 (7.6)	1.17 (0.81, 1.70)	
Physical activity, kcal/wk†			0.20*
<500	1148 (52.3)		
500–1500	598 (27.3)	0.96 (0.74, 1.23)	
≥1500	447 (20.4)	1.18 (0.91, 1.54)	
Hypertension‡	1258 (57.4)	1.28 (1.03, 1.59)	0.02
Diabetes mellitus	292 (13.3)	1.63 (1.24, 2.13)	<0.001
Body mass index, kg/m^2^	27.41±4.91	1.02 (1.00, 1.04)	0.10
Abdominal circumference	99.43±13.54	1.01 (1.00, 1.02)	0.02
Systolic blood pressure, per 10 mmHg	135.72±20.63	1.09 (1.04, 1.15)	<0.001
Diastolic blood pressure, per 10 mmHg	71.59±11.66	1.12 (1.02, 1.22)	0.01
Total cholesterol, per 10 mg/dl	204.83±37.93	0.99 (0.96, 1.01)	0.31
HDL-cholesterol, per 10 mg/dl	55.46±17.12	0.87 (0.81, 0.93)	<0.001
Total/HDL-cholesterol	3.98±1.22	1.15 (1.06, 1.25)	<0.001
LDL-cholesterol, per 10 mg/dl	122.87±34.44	1.01 (0.98, 1.04)	0.50
Triglycerides, mg/dl∥	116 (87–160)	1.11 (0.89, 1.39)	0.36
Glucose, per 10 mg/dl	102.46±31.88	1.05 (1.03, 1.08)	<0.001
Framingham risk score, %¶			<0.001
<5%	468 (21.3)		
5–9.99%	557 (25.4)	1.35 (0.92, 1.99)	
10–19.99%	543 (24.8)	2.12 (1.47, 3.04)	
≥20%	625 (28.5)	3.06 (2.17, 4.31)	
Creatinine, mg/dl∥	1 (0.9–1.1)	1.96 (1.33, 2.87)	0.001
GFR, ml/min/1.73 m^2^ #	61.15±15.05	1.00 (0.99, 1.01)	0.87
GFR (categories)**			0.63
≥80	525 (23.9)	1.00	
70–79.99	536 (24.5)	1.11 (0.83,1.49)	
60–69.99	555 (25.3)	0.87 (0.64,1.18)	
<60	576 (26.3)	1.00 (0.75,1.35)	
Medication use			
Lipid-lowering	229 (10.4)	1.04 (0.75, 1.46)	0.79
Ace inhibitors	273 (12.4)	1.14 (0.84, 1.54)	0.39
Hormone replacement therapy	48 (2.2)	0.82 (0.37, 1.85)	0.64
Aspirin	412 (18.8)	1.31 (1.02, 1.69)	0.03

Abbreviations: SD: standard deviation; HR: hazard ratio; CI: confidence interval; HDL: high-density lipoprotein; LDL: low-density lipoprotein; GFR: glomerular filtration rate.

*p for trend.

† Physical activity was assessed by questionnaire about all types of walking and exercise performed in the prior week [14].

‡ Defined by self-report of hypertension and use of anti-hypertensive medications, or measured SBP≥140 and/or DBP≥90 mmHg.

∥ Expressed as median (25%–75%), because of skewed distribution. The effect of the logarithm of the covariates on the CHD is measured.

¶ Classes of CHD risk at 10 years, according to Framingham functions [2].

# Glomerular filtration rate (GFR) was estimated using the MDRD equation: GFR = 175 * Creatinine^−1.154^ * Age^−0.203^ * (1.212*I_black_+I_white_)* (0.742*I_female_+I_men_) [32].

**Quartiles were used instead of clinical cut-offs to avoid categories with few participants. In particular, categories of GFR<15 and within 15–29.99 were collapsed with the category 30–59.99 (only 0.2% in the class of GFR<15 and 0.5% in the class of GFR within 15–29.99) and a ≥80 category was replaced to the usual ≥90 (only 7% for GFR≥90).

During a median follow-up of 8.3 years (maximum, 10.2 years), 351 participants developed a CHD event (197 of which had a “hard” CHD event). In unadjusted analyses, all traditional cardiovascular risk factors were associated with CHD events except for total cholesterol and LDL-cholesterol (Table 1). Abdominal circumference, glucose, and creatinine were also associated with CHD events, but not glomerular filtration rate, alcohol use or physical activity levels. Results were similar for hard CHD events with larger confidence intervals because of lower number of events (data not shown), except that the association with abdominal circumference disappeared (HR = 1.00).

**Figure 1:**
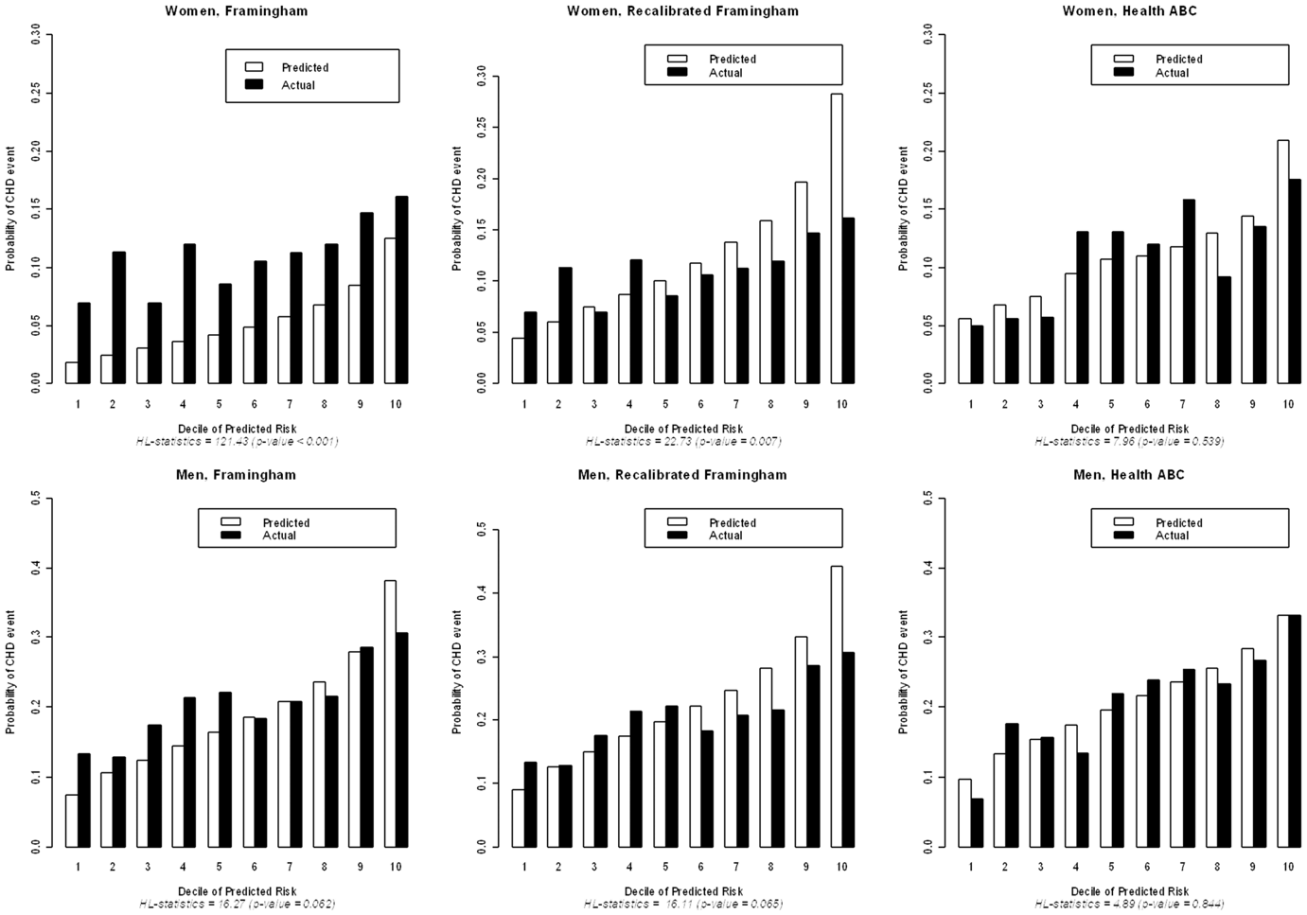
Predicted risk of CHD events at 7.5 years according to original Framingham functions, recalibrated Framingham functions and Health ABC functions.

Number of participants in different risk factor categories and CHD events are shown in Table 2 for women and Table 3 for men. The original FRS had poor discrimination in these older adults (C-index: 0.577 in women; 0.583 in men). Using risk factors as continuous variables yielded similar C-indexes. Calibration of the original FRS was also poor in older adults (Figure 1), particularly among women, for whom the absolute risk was underestimated by 51% (vs. 8% in men, Table S1). Recalibration of the FRS improved calibration, particularly for women, and produced a better match between observed and expected CHD risk (Figure 1, Table S1). Statistically significant differences between observed and expected risks across deciles remained, at least in in women (p value remaining <0.05, larger p-values indicating better calibration), with an overestimation of the predicted risk for those above the median risk by a factor of 1.4 for women and 1.3 for men. For both genders, the Health ABC function significantly improved calibration (Figure 1). For discrimination, the C-index for the Health ABC function, after correction for optimism, was comparable to the C-index of the FRS (p = 0.54 for women and 0.90 for men, Tables 2 and 3). Total cholesterol and age^2^ were not predictive in women and were therefore omitted in the Health ABC function. Overall, results did not differ between whites and blacks. C-indexes for the unmodified FRS, the recalibrated FRS and the Health ABC function stratified by gender did not significantly differ between whites and blacks (all p for interaction >0.20; C-indexes ranging from 0.550 to 0.603). Calibration became reasonable (with p>0.20 for comparison of observed with expected) in white men for the unmodified FRS and in white men and women for the recalibrated FRS, but the best calibration remained for the Health ABC function (p>0.20 for comparison of observed with expected in the four subgroups stratified by race and gender).

**Table 2:** Discrimination and calibration of Framingham functions (FRS), recalibrated FRS and Health ABC function in women (n = 1212).

	Participants with Risk Factor, N (%)	CHD events (N)	FRS	Recalibrated FRS	Refit FRS (Health ABC function )*	
			Coef†	Coef†	Coef (95%CI)	HR (95% CI)
Age, y, mean (SD)	73.41 (2.84)	146	0.33766	0.33766	0.00 (−0.06,0.06)	1.00 (0.95,1.06)
Age^2^			−0.00268	−0.00268		
TC, mg/dL‡						
<160	78 (6%)	11	−0.26138	−0.26138		
160–199	364 (30%)	46	Referent	Referent		
200–239	496 (41%)	52	0.20771	0.20771		
240–279	217 (18%)	29	0.24385	0.24385		
≥280	57 (5%)	8	0.53513	0.53513		
HDL-C, mg/dL‡						
<35	21 (2%)	2	0.84312	0.84312	0.21 (−0.30,0.71)	1.23 (0.74 ,2.04)
35–44	149 (12%)	22	0.37796	0.37796		
45–49	149 (12%)	21	0.19785	0.19785	0.14 (−0.39,0.67)	1.15 (0.68,1.95)
50–59	322 (27%)	41	Referent	Referent	Referent	Referent
≥60	571 (47%)	60	−0.42951	−0.42951	−0.10 (−0.51,0.30)	0.90 (0.60,1.35)
Blood pressure∥						
Optimal	266 (22%)	19	−0.53363	−0.53363	−0.26 (−0.86,0.33)	0.77 (0.42,1.40)
Normal	259 (21%)	25	Referent	Referent	Referent	Referent
High normal	254 (21%)	38	−0.06773	−0.06773	0.41 (−0.09,0.92)	1.51 (0.91,2.51)
Stage I hypertension	296 (24)	46	0.26288	0.26288	0.45 (−0.02,0.91)	1.56 (0.98,2.49)
Stage II–IV hypertension	137 (11%)	18	0.46573	0.46573		
Diabetes	141 (12%)	27	0.59626	0.59626	0.62 (0.20,1.05)	1.86 (1.22,2.85)
Smoker						
Never	714 (59%)	83	Referent	Referent	Referent	Referent
Former	388 (32%)	48				
Current	110 (9%)	15	0.29246	0.29246	0.29 (−0.25,0.83)	1.34 (0.78,2.29)
Mean survival function at t = 7.5 years, S_0_(t)			0.9717¶	0.8898#	0.8962**	
C-index			0.577	0.577	0.598††	
H-L statistics∥ ∥			121.43 (<0.001)	22.73 (0.007)	7.96 (0.539)	

Abbreviations: FRS: Framingham risk score; CHD: coronary heart disease; coef: coefficient; CI: confidence interval; SD: standard deviation; TC: total cholesterol; HDL-C: high-density lipoprotein cholesterol.

*Some of the Framingham risk factors categories were collapsed to avoid cells with limited numbers of events and /or unpredictive trends. Total cholesterol and age^2^ were omitted because they were unpredictive in these older women. The proportionality assumption was tested using the Therneau and Grambsch statistics, which is based on the Schoenfeld residuals. The assumption was accepted (p = 0.14).

† Based on Wilson et al. [2].

‡ Cholesterol categories proposed by the National Cholesterol Education Program [24].

∥ Blood pressure categories: Optimal (Systolic<120, Diastolic>80); Normal (Systolic<130, Diastolic>85); High normal (Systolic<140, Diastolic>90); Stage I (Systolic<160, Diastolic<100); Stage II–IV (Systolic ≥160, Diastolic ≥100) [24].

¶ Estimated from the Framingham adjusted survival rate (survival rate at the mean value of the risk factors) at 10 years: S_0_(10) = 0.96246 [2], as: Ŝ_0_(7.5) = S_0_(10)^0.75^ = 0.9717 (exponential model).

# Kaplan-Meier survival function at t = 7.5 years on HABC data, similar to reference [24].

**Adjusted survival rate at t = 7.5 years obtained on the HABC cohort as the baseline survival functions of the multivariate Cox model, similar to reference [9].

†† After bootstrap correction for the optimism (1000 bootstrap samples from the original dataset [20]), c-index = 0.564 (p = 0.54 for comparison with Framingham function).

∥ ∥ Adaptation to the Cox model of the Hosmer-Lemeshow test of goodness of fit [33], comparing observed and expected failures within deciles of predicted risk. Larger p values indicate better calibration [21].

**Table 3:** Discrimination and calibration of Framingham functions (FRS), recalibrated FRS and Health ABC function in men (n = 981).

	Participants with Risk Factor, N (%)	CHD events (N)	FRS	Recalibrated FRS	Refit FRS (Health ABC function )*	
			Coef†	Coef†	Coef (95% CI)	HR (95% CI)
Age, y, mean (SD)	73.613 (2.86)	205	0.04826	0.04826	0.05 (−0.00,0.10)	1.05 (1.00,1.10)
TC, mg/dL‡						
<160	139 (14%)	23	−0.65945	−0.65945	−0.32 (−0.78,0.14)	0.73 (0.46,1.15)
160–199	451 (46%)	94	Referent	Referent	Referent	Referent
200–239	303 (31%)	69	0.17692	0.17692		
240–279	70 (7%)	15	0.50539	0.50539	0.10 (−0.20,0.39)	1.10 (0.82,1.48)
≥280	18 (2%)	4	0.65713	0.65713		
HDL-C, mg/dL‡						
<35	140 (14%)	28	0.49744	0.49744		
35–44	295 (30%)	72	0.24310	0.24310	Referent	Referent
45–49	160 (16%)	38	Referent	Referent		
50–59	204 (21%)	40	−0.05107	−0.05107	−0.23 (−0.58,0.13)	0.80 (0.56,1.13)
≥60	182 (19%)	27	−0.48660	−0.48660	−0.60 (−1.02 ,−0.19)	0.55 (0.36,0.83)
Blood pressure∥						
Optimal	214 (22%)	27	−0.00226	−0.00226	−0.47 (−0.95,0.02)	0.63 (0.39,1.02)
Normal	210 (22%)	42	Referent	Referent	Referent	Referent
High normal	188 (19%)	51	0.28320	0.28320		
Stage I hypertension	258 (26%)	59	0.52168	0.52168	0.18 (−0.16,0.53)	1.20 (0.85,1.70)
Stage II–IV hypertension	111 (11%)	26	0.61859	0.61859		
Diabetes	151 (15%)	38	0.42839	0.42839	0.23 (−0.12,0.58)	1.26 (0.88,1.79)
Smoker						
Never	302 (31%)	56	Referent	Referent	Referent	Referent
Former	568 (58%)	125				
Current	111 (11%)	24	0.52337	0.52337	0.28 (−0.15,0.71)	1.32 (0.86,2.03)
Mean survival function at t = 7.5 years, S_0_(t)			0.9241¶	0.7929#	0.8032**	
C-index			0.583	0.583	0.606††	
H-L statistics∥ ∥			16.27 (0.062)	16.11 (0.065)	4.89 (0.844)	

Abbreviations: FRS: Framingham risk score; CHD: coronary heart disease; coef: coefficient; CI: confidence interval; SD: standard deviation; TC: total cholesterol; HDL-C: high-density lipoprotein cholesterol.

*Some of the Framingham risk factors categories were collapsed to avoid cells with limited numbers of events and /or unpredictive trends. The proportionality assumption was tested using the Therneau and Grambsch statistics, which is based on the Schoenfeld residuals. The assumption was accepted (p = 0.33).

† Based on Wilson et al. [2].

‡ Cholesterol categories proposed by the National Cholesterol Education Program [24].

∥ Blood pressure categories: Optimal (Systolic<120, Diastolic>80); Normal (Systolic<130, Diastolic>85); High normal (Systolic<140, Diastolic>90); Stage I (Systolic<160, Diastolic<100); Stage II–IV (Systolic ≥160, Diastolic ≥100) [24].

¶ Estimated from the Framingham adjusted survival rate (survival rate at the mean value of the risk factors) at 10 years: S_0_(10) = 90015 [2], as: Ŝ_0_(7.5) = S_0_(10)^0.75^ = 0.9241 (exponential model).

# Kaplan-Meier survival function at t = 7.5 years on HABC data, similar to reference [24].

**Adjusted survival rate at t = 7.5 years obtained on the HABC cohort as the baseline survival functions of the multivariate Cox model, similar to reference [9].

†† After bootstrap correction for the optimism (1000 bootstrap samples from the original dataset [20]), c-index = 0.580 (p = 0.90 for comparison with Framingham function).

∥ ∥ Adaptation to the Cox model of the Hosmer-Lemeshow test of goodness of fit [33], comparing observed and expected failures within deciles of predicted risk. Larger p values indicate better calibration [21].

We used a variety of model selection procedures when considering the addition of routinely available measures not included in the Framingham risk factor set to the Health ABC function. The procedures based on p-values and the AIC lead to very similar final models (Table S2); in contrast, the BIC, which strongly penalizes the complexity of the model, lead to the omission of a larger number of risk factors. All final models mainly retained traditional risk factors included in the FRS. The additions of lifestyle variables (alcohol, physical activity), waist circumference, and creatinine did not improve risk prediction in terms of discrimination or model fit beyond using the traditional risk factors from the FRS. Selection procedures stratified by gender yielded similar results.

## Discussion

In this population-based study of older adults, the FRS poorly discriminated between persons who experienced a CHD event and those who did not (C-index: 0.577 in women; 0.583 in men) and underestimated the absolute CHD risk by 51% in women and 8% in men. Nevertheless, traditional risk factors remained the best predictors of CHD events. Physical activity, alcohol consumption, waist circumference and creatinine did not improve risk prediction beyond traditional risk factors of the FRS. Recalibration of the FRS improved the accuracy of absolute risk estimation, particularly for women. For both genders, the Health ABC function significantly improved estimation of absolute risk, with a discrimation similar to the FRS. Neither refitting equations nor including other routinely available measurements in risk equations provided substantial benefits in terms of discriminating between high- and low-risk older adults over FRS.

Our study adds new data on the performance of recalibration of the FRS, refit functions and the utility of adding other routinely available risk parameters to FRS among older adults. Previous studies also found lower performance of risk prediction based on the FRS associated with increasing age, but did not examine how CHD risk prediction might be improved among older adults. For example, the C-index for the FRS was 0.63/0.66 in men/women aged 65–74 enrolled in the Cardiovascular Health Study [24] and 0.63 in a patient cohort with a mean age of 66 years [25], compared to 0.79/0.83 in men/women enrolled in the Framingham Heart Study (mean age of 49 years) [24]. Performance of the FRS may be worse in the very old, with a C-index of 0.53 in adults aged 85 years or older [26]. In different ethnic populations in the US and other countries, FRS often overestimates CHD risk [9], [24], [27]. Recalibration of the FRS was shown to improve the estimation of absolute risk in these different ethnic populations [9], [24]. In the present analysis among older adults, the FRS underestimated absolute CHD risk, particularly in women. Although recalibration of the FRS yielded a better estimation of absolute risk, the function specific to the Health ABC cohort yielded the best estimation of absolute risk, becoming statistically acceptable. Compared to recalibration among other ethnic groups [9], [24], the recalibrated FRS showed worse risk prediction in our study of older adults. Our results indicate that the FRS not only underestimates CHD risk in older adults but that some traditional risk factors, such as total and LDL-cholesterol, have weaker associations with CHD risk in older adults, as previoulsy found [7]. In particular, total cholesterol did not predict CHD events in older women in our present study.

Our study has several strengths and limitations. These data are drawn from a well-characterized population-based cohort of older adults, with a high number of CHD events over a 8-year follow-up period, and included a larger sample of black older adults compared to previous studies [24]. CHD events were formally adjudicated. The cohort included both white and black older adults, but did not include other ethnic groups. After stratification by gender, our power for subgroup analyses was limited for comparisons between whites and blacks. Lower performance of the FRS might partly be related to ascertainment of CHD events limited to those requiring hospitalization in the Health ABC, but not in the Framingham cohort [2]. However, all our comparisons in the present data examined CHD outcomes limited to those requiring hospitalization; we also found similar associations for hard CHD events (nonfatal myocardial infarction or coronary death).

What are the potential clinical and research implications of these findings? Clinicians should use the FRS with caution in older adults, as it underestimates the absolute CHD risk by 51% in women and 8% in men and does not discriminate effectively between those who will have CHD events and those who will not. We could not identify additional, routinely available variables that might improve risk prediction beyond traditional risk factors comprising the FRS, similar to several previous studies that did not clearly identify factors improving risk prediction of the FRS [28]. Re-estimated risk functions using these factors improve accurate estimation of absolute risk, but did not meaningfully improve discrimination, or the ability to distinguish between low, intermediate, and high-risk adults. Substantial improvements in discrimination may require novel CHD risk markers or other strategies for risk prediction in the elderly. We have previously found that ankle-arm index and interleukin-6, but not high-sensitive C-reactive protein, improved risk prediction beyond traditional risk factors, but only modestly [17]. Other potential markers that might improve CHD risk prediction in the elderly include homocysteine [26] or coronary calcification [29]. Future investigations should examine whether markers of atherosclerosis [29] or novel CHD risk markers [30] might improve risk prediction beyond FRS in older adults, which still requires additional studies [31]. For current clinical use, recalibrated Framingham functions seem an attractive option to better assess absolute CHD risk for older adults (Methods S1), given that no currently available new risk factors have been clearly and consistently shown to improve CHD risk prediction [28] and that the Health ABC function needs to be externally validated in another cohort.

In summary, our study suggests that the FRS underestimates CHD risk in the growing population of elderly [8], particularly in older women. However, traditional risk factors remain the best predictors of future CHD events. Recalibrating risk functions in older adults is important to improve the accuracy of absolute CHD risk estimates, especially for women, and might be useful to better identify older individuals at increased risk who will benefit from preventive therapies, such as statins or aspirin. However, substantial improvements in discrimination may require novel CHD risk markers or other strategies for better CHD risk prediction and risk stratification in the elderly.

## Supporting Information

Table S1 Ratio of predicted to observed risks for original Framingham functions (FRS), recalibrated FRS and Health ABC functions across deciles of predicted risk.
(DOCX)

Table S2 Independent predictors of CHD according to different strategies of model selection (n = 2193).
(DOCX)

Methods S1 (DOCX)
