# Supplementary material for: Framingham Risk Score and Alternatives for Prediction of Coronary Heart Disease in Older Adults
Source: PLoS One. 2012 Mar 28;7(3):e34287. doi: 10.1371/journal.pone.0034287 (PMC3314613; doi:10.1371/journal.pone.0034287)
Supplement: Table S1 — Ratio of predicted to observed risks for original Framingham functions (FRS), recalibrated FRS and Health ABC functions across deciles of predicted risk. (DOCX) [file pone.0034287.s001.docx]

**Table S1. Ratio of predicted to observed risks for original Framingham functions (FRS), recalibrated FRS and Health ABC functions across deciles of predicted risk.**

|  | **FRS** | | |  | **Recalibrated FRS** | | |  | **Refit FRS (Health ABC function )** | | |
| --- | --- | --- | --- | --- | --- | --- | --- | --- | --- | --- | --- |
| **Deciles of risk** | **Predicted risk (%)** | **Observed risk (%)** | **Ratio** |  | **Predicted risk (%)** | **Observed risk (%)** | **Ratio** |  | **Predicted risk (%)** | **Observed risk (%)** | **Ratio** |
| **Women:** |  |  |  |  |  |  |  |  |  |  |  |
| First | 1.82 | 6.98 | 0.26 |  | 4.44 | 6.98 | 0.64 |  | 5.61 | 4.94 | 1.14 |
| Second | 2.48 | 11.31 | 0.22 |  | 6.03 | 11.31 | 0.53 |  | 6.78 | 5.61 | 1.21 |
| Third | 3.10 | 6.96 | 0.45 |  | 7.52 | 6.96 | 1.08 |  | 7.47 | 5.71 | 1.31 |
| Fourth | 3.60 | 12.02 | 0.30 |  | 8.70 | 12.02 | 0.72 |  | 9.49 | 13.08 | 0.73 |
| Fifth | 4.18 | 8.58 | 0.49 |  | 10.06 | 8.58 | 1.17 |  | 10.73 | 13.07 | 0.82 |
| Sixth | 4.91 | 10.57 | 0.46 |  | 11.75 | 10.57 | 1.11 |  | 10.98 | 11.99 | 0.92 |
| Seventh | 5.79 | 11.28 | 0.51 |  | 13.76 | 11.28 | 1.22 |  | 11.80 | 15.85 | 0.74 |
| Eighth | 6.76 | 11.94 | 0.57 |  | 15.93 | 11.94 | 1.33 |  | 12.95 | 9.18 | 1.41 |
| Ninth | 8.45 | 14.71 | 0.57 |  | 19.65 | 14.71 | 1.34 |  | 14.42 | 13.55 | 1.06 |
| Tenth | 12.56 | 16.10 | 0.78 |  | 28.19 | 16.10 | 1.75 |  | 20.96 | 17.54 | 1.19 |
| **Overall** | **5.36** | **11.02** | **0.49** |  | **12.60** | **11.02** | **1.14** |  | **11.09** | **11.02** | **1.01** |
| **Men:** |  |  |  |  |  |  |  |  |  |  |  |
| First | 7.53 | 13.37 | 0.56 |  | 9.10 | 13.37 | 0.68 |  | 9.75 | 6.80 | 1.43 |
| Second | 10.57 | 12.82 | 0.82 |  | 12.74 | 12.82 | 0.99 |  | 13.30 | 17.64 | 0.75 |
| Third | 12.45 | 17.52 | 0.71 |  | 14.97 | 17.52 | 0.85 |  | 15.39 | 15.74 | 0.98 |
| Fourth | 14.52 | 21.31 | 0.68 |  | 17.42 | 21.31 | 0.82 |  | 17.51 | 13.44 | 1.30 |
| Fifth | 16.48 | 22.14 | 0.74 |  | 19.72 | 22.14 | 0.89 |  | 19.62 | 21.91 | 0.90 |
| Sixth | 18.54 | 18.32 | 1.01 |  | 22.13 | 18.32 | 1.21 |  | 21.74 | 23.85 | 0.91 |
| Seventh | 20.76 | 20.71 | 1.00 |  | 24.71 | 20.71 | 1.19 |  | 23.57 | 25.39 | 0.93 |
| Eighth | 23.70 | 21.57 | 1.10 |  | 28.10 | 21.57 | 1.30 |  | 25.62 | 23.26 | 1.10 |
| Ninth | 28.00 | 28.60 | 0.98 |  | 33.01 | 28.60 | 1.15 |  | 28.40 | 26.84 | 1.06 |
| Tenth | 38.19 | 30.59 | 1.25 |  | 44.32 | 30.59 | 1.45 |  | 33.14 | 33.26 | 1.00 |
| **Overall** | **19.03** | **20.71** | **0.92** |  | **22.57** | **20.71** | **1.09** |  | **20.75** | **20.71** | **1.00** |
