# Supplementary material for: Framingham Risk Score and Alternatives for Prediction of Coronary Heart Disease in Older Adults
Source: PLoS One. 2012 Mar 28;7(3):e34287. doi: 10.1371/journal.pone.0034287 (PMC3314613; doi:10.1371/journal.pone.0034287)
Supplement: Table S2 — Independent predictors of CHD according to different strategies of model selection (n = 2193). (DOCX) [file pone.0034287.s002.docx]

# Table S2. Independent predictors of CHD according to different strategies of model selection (n=2193)

|  | All * | | | p <0.10 † | | AIC ‡ | | | BIC \|\| | | |
| --- | --- | --- | --- | --- | --- | --- | --- | --- | --- | --- | --- |
|  | Coef | p | Coef | | p | | Coef | p | | Coef | P |
| Age ¶ | 0.03 | (0.12) | 0.03 | | (0.11) | | 0.03 | (0.13) | | 0.02 | (0.20) |
| Women | -0.50 | (0.00) | -0.48 | | (0.00) | | -0.48 | (0.00) | | -0.6 | (0.00) |
| Current smoker | 0.38 | (0.04) | 0.36 | | (0.05) | | 0.38 | (0.04) | |  |  |
| Former smoker | 0.19 | (0.11) | 0.21 | | (0.09) | | 0.19 | (0.11) | |  |  |
| Hypertension | 0.07 | (0.63) |  | |  | |  |  | |  |  |
| Diabetes | 0.36 | (0.01) | 0.38 | | (0.01) | | 0.36 | (0.01) | | 0.38 | (0.01) |
| Waist circumference. | 0.00 | (0.28) |  | |  | | 0.00 | (0.26) | | 0.01 | (0.14) |
| Systolic BP | 0.08 | (0.02) | 0.09 | | (0.00) | | 0.09 | (0.00) | | 0.08 | (0.00) |
| HDL-C | -0.07 | (0.07) | -0.08 | | (0.04) | | -0.06 | (0.09) | |  |  |
| TC | 0.01 | (0.39) |  | |  | |  |  | |  |  |
| Creatinine # | 0.01 | (0.98) |  | |  | |  |  | |  |  |
| LR test (df) | 67.4 (11) | 3.8*10^(-10)^ | 66.8 (7) | | 6.6*10^(-12)^ | | 66.6 (8) | 2.4*10^(-11)^ | | 58.9 (5) | 2.0*10^(-11)^ |
| AIC | 5171.1 | | 5181.4 | | | | 5166.3 | | | 5167.9 | |
| BIC | 5217.4 | | 5212.3 | | | | 5201.0 | | | 5191.0 | |
| C-index ** | 0.631 | | 0.630 | | | | 0.630 | | | 0.620 | |

Abbreviations: AIC: Akaike information criterion; BIC: Bayesian information criterion; coef: coefficient; BP: blood pressure; HDL-C: high-density lipoprotein cholesterol; TC: total cholesterol; LR: likelihood ratio

* The « All » model contains all variables displaying a p <0.2 in the bivariate models (Table 1) and age, except glucose and BMI to avoid collinearity with diabetes and waist circumference.

† Backward deletion based on p <0.10.

‡ Forward selection using Akaike information criterion (AIC)[33]: a likelihood-based measure in which a simple measure of goodness of fit (-2 times the log-likelihood) is penalized for the number of predictors in the model. A lower value indicates better prediction.

|| Forward selection using Bayes Information Criterion (BIC)[21]. Similar to AIC, this measure of predictiveness imposes a more severe penalty for the number of predictors.  Lower values indicate better prediction.

¶ Age was forced to enter all the final models

# Creatinine was examined as logarithm of creatinine.

** Harrell’s C-index, an adaptation to survival models of the C-statistic (equivalently, the area under the ROC curve) in logistic models. Higher values indicate better discrimination. After bootstrap correction for the optimism (1000 bootstrap samples from the original dataset), C-index = 0.615 (“All” model); C-index = 0.621 (“p<0.10” model); C-index = 0.620 (“AIC” model) and C-index = 0.614 (“BIC” model). For separate models by gender, all C-indexes were lower.
