# Supplementary material for: Framingham Risk Score and Alternatives for Prediction of Coronary Heart Disease in Older Adults
Source: PLoS One. 2012 Mar 28;7(3):e34287. doi: 10.1371/journal.pone.0034287 (PMC3314613; doi:10.1371/journal.pone.0034287)
Supplement: Methods S1 — (DOCX) [file pone.0034287.s003.docx]

**Application of Tables 2 and 3 to an individual patient**

Take as an example a male subject, 73 years old, with a total cholesterol of 196 mg/dL, an HDL cholesterol of 41 mg/dL, high normal blood pressure, nonsmoking, with diabetes. In the following, we will calculate his CHD risk at 7.5 years according to the different functions presented in the Method Section.

**Framingham Functions (F):**

R_(F)_= 1-S_(F)_(7.5) ^R(F)^ = 0.26933

With S_(F)_(7.5) = 0.9241 (Table 3); and R_(F)_ = exp[0.04826*(73-M_1_) - 0.65945*(0-M_2_) + 0.17692*(0-M_3_) + 0.50539*(0-M_4_) + 0.65713*(0-M_5_) + 0.49744*(0-M_6_) + 0.24310*(1- M_7_) - 0.05107*(0-M_8_) - 0.48660*(0-M_9_) -0.00226*(0-M_10_) + 0.28320*(1-M_11_) + 0.52168*(0-M_12_) + 0.61859*(0-M_13_) + 0.42839*(1-M_14_) + 0.52337*(0-M_15_)] ; being (M_1_, M_2_, … , M_15_) the mean values of risk factors (ordered as in Table 3) in the Framingham cohort. These means can be found in the Appendix of Wilson et al[2]: (M_1_, M_2_, … , M_15_) = (48.5926, 0.07433, 0.38851, 0.16673, 0.05826, 0.19285, 0.35476, 0.19646, 0.10727, 0.20048, 0.20048, 0.22820, 0.13057, 0.05223, 0.40458)

**Recalibrated Framingham Functions (rF):**

R_(rF)_= 1-S_(rF)_(7.5) ^R(rF)^ = 0.31442

With S_(rF)_(7.5) = 0.7929 (Table 3); and R_(rF)_ = exp[0.04826*(73-N_1_) - 0.65945*(0-N_2_) + 0.17692*(0-N_3_) + 0.50539*(0-N_4_) + 0.65713*(0-N_5_) + 0.49744*(0-N_6_) + 0.24310*(1- N_7_) - 0.05107*(0-N_8_) - 0.48660*(0-N_9_) -0.00226*(0-N_10_) + 0.28320*(1-N_11_) + 0.52168*(0-N_12_) + 0.61859*(0-N_13_) + 0.42839*(1-N_14_) + 0.52337*(0-N_15_)] ; being (N_1_, N_2_, … , N_15_) the mean values of risk factors in the Health ABC cohort. These means are: (N_1_, N_2_, … , N_15_) = (73.61264, 0.14169 0.30887, 0.07136, 0.01835, 0.14271, 0.30071, 0.20795, 0.18552, 0.21814, 0.19164, 0.26300, 0.11315, 0.15392, 0.11315)

**Health ABC functions (H):**

R_(H)_= 1-S_(H)_(7.5) ^R(H)^ = 0.29844

With S_(H)_(7.5) = 0.8032 (Table 3); and R_(H)_ = exp[0.04809*(73-Q_1_) - 0.31653*(0- Q_2_) + 0.09636*(0- Q_3_) + 0.22675*(0- Q_4_) - 0.60452*(0- Q_5_) - 0.46745*(0- Q_6_) + 0.18442*(1- Q_7_) + 0.22972*(1- Q_8_) 0.27851*(0- Q_9_)] ; being (Q_1_, Q_2_, … , Q_9_) the mean values of risk factors with collapsed categories in the Health ABC cohort. These means are: (Q_1_, Q_2_, … , Q_9_) = (73.61264, 0.14169, 0.39857, 0.20795, 0.18552, 0.21814, 0.56779, 0.15392, 0.11315).
